# Efficacy and Safety of a Multistrain Probiotic Formulation Depends from Manufacturing

**DOI:** 10.3389/fimmu.2017.01474

**Published:** 2017-11-06

**Authors:** Vito Trinchieri, Luca Laghi, Beatrice Vitali, Carola Parolin, Ilaria Giusti, Daniela Capobianco, Paola Mastromarino, Claudio De Simone

**Affiliations:** ^1^Department of Public Health and Infectious Diseases, Sapienza University, Rome, Italy; ^2^Department of Agricultural and Food Sciences, Interdepartmental Centre for Agri-Food Industrial Research, University of Bologna, Cesena, Italy; ^3^Department of Pharmacy and Biotechnology, University of Bologna, Bologna, Italy; ^4^Department of Life, Health and Environmental Sciences, University of L’Aquila, Piazzale S. Tommasi, Coppito, Italy; ^5^Department of Public Health and Infectious Disease, Section of Microbiology, Sapienza University Rome, Rome, Italy; ^6^Chateau d’Oex, Switzerland

**Keywords:** human immunodeficiency virus, probiotics, metabolomics, gut, microbiota, VSL#3

## Abstract

**Background:**

Variability in probiotics manufacturing may affect their properties, with potential implications for their efficacy and safety. This is of particular concern with probiotic products destined for use in patients with serious medical conditions, including human immunodeficiency virus (HIV) infection. The purpose of the study was to carry out a series of experiments comparing the properties of the US-made probiotic formulation originally commercialized under the brand name VSL#3^®^, with those of the Italian-made formulation now commercialized under the same name. The US-made formulation has previously shown beneficial effects at the intestinal and neurological levels in HIV-infected subjects as well as in patients with inflammatory bowel diseases and hepatic encephalopathy.

**Methods:**

Eleven subjects receiving combined antiretroviral therapy for HIV-1 were treated for 6 months with the US-made VSL#3 formulation. At baseline and 6 months, T-cells were analyzed for phenotype and activation markers, and fecal samples were analyzed for bifidobacteria, lactobacilli, and their metabolites. The fecal metabolome was assessed using ^1^H-NMR spectroscopy. Production of metabolites of interest by bacteria obtained from sachets of the two formulations was compared *in vitro* and their effects on a rat intestinal epithelial cell line (IEC-6) were assessed. Particular attention was paid to the metabolite 1,3-dihydroxyacetone (DHA).

**Results:**

At 6 months, fecal samples showed a significant increase in the specific bacterial genera contained in the probiotic supplement. Immune activation was reduced as shown by a significant reduction in the percentage of CD4^+^CD38^+^HLA-DR^+^ T-cells at 6 months. Fecal concentrations of DHA decreased significantly. *In vitro*, significant differences in the production and metabolism of DHA were found between bacteria from the US-made and Italian-made formulations: the US-made formulation was able to metabolize DHA whereas the bacteria in the Italian-made formulation were producing DHA. DHA reduced the viability of *Streptococcus thermophilus*, reduced IEC-6 cell viability in a dose-dependent manner, and also led to a lower rate of repair to scratched IEC-6 cell monolayer.

**Conclusion:**

Our data, in conjunction with previously published findings, confirm that the new Italian-made formulation of VSL#3^®^ is different from the previous US-made VSL#3 and therefore its efficacy and safety in HIV-infected subjects is still unproven.

## Introduction

Variability in the manufacturing of probiotics is an unexplored area that is of major concern for efficacy and safety, especially if the product is destined for use by individuals affected by serious conditions such as human immunodeficiency virus (HIV), inflammatory bowel disease (IBD), or cancer. Since bacterial gene expression is heavily affected by the growth media and by industrial processing parameters, modifications in the manufacturing unit, reagents, and know-how used can make the bacterial strains more or less “probiotic” in their properties (1). The immunological and biochemical profile of the final product can be further modified by the fact that the bacteria are incorporated into numerous carrier matrices, which influence the bacterial metabolic pathways, especially short-chain fatty acid production and tryptophan metabolism (2, 3).

In the specific case of benefits from probiotic treatment observed in the intestinal tract and the central nervous system in patients receiving combined antiretroviral therapy (cART), the probiotic formulation produced at Dupont/Danisco in the US and containing the strains *Lactobacillus plantarum* DSM24730, *Streptococcus thermophilus* DSM24731, *Bifidobacterium breve* DSM24732, *Lactobacillus paracasei* DSM24733, *Lactobacillus delbrueckii subsp. bulgaricus* DSM24734, *Lactobacillus acidophilus* DSM 24735, *Bifidobacterium longum* DSM24736, *Bifidobacterium infantis* DSM24737, can currently be considered the “reference formulation” because it is the formulation with which the most convincing efficacy and safety data were obtained (2–4). The human data are supported by observations in monkeys experimentally infected with Simian immunodeficiency virus (SIV) (5–7) and the same formulation is under evaluation in the multicenter trial ACTG A5350. However, this formulation is no longer commercially available under the brand name VSL#3^®^ in Europe, Canada, or some other countries, and the brand name is now applied to a formulation manufactured at CSL/Nutrilinea in Italy. Because it comes from a different source, this new formulation, although it is commercialized under the same VSL#3^®^ trademark, might be not interchangeable with the formulation on which the efficacy and safety data are based.

When genetic or production changes occur, the need for reassessing efficacy and safety is mandatory. Unfortunately, while we have assays to understand probiotic physiology, the methods and tests causally linked to probiotic efficacy/safety in specific diseases are lacking. In this paper, we describe a new approach characterized by (a) the identification of the metabolites present in the feces of cART patients who had received 6 months’ treatment with the “reference” (US-made) formulation, and then (b) the *in vitro* comparison for production and metabolism of the same molecules by the US-made (the formulation administered to cART patients) and the new Italian-made VSL#3, and (c) their biological effects on a normal rat intestinal epithelial cell line. With this approach, rather than measuring probiotic viability at consumption and at excretion, we evaluated probiotic function at the site of action and identified some mechanisms by which genetic or physiological changes could affect the efficacy/safety of the formulation.

## Materials and Methods

The study was approved by the institutional review board (Department of Public Health and Infectious Diseases, Sapienza University of Rome; and the Ethics Committee of Umberto I General Hospital, Rome, protocol number 2970). All study participants signed written informed consent.

### Clinical Study

#### Participants and Interventions

Eleven HIV-1 positive patients treated with cART and virologically suppressed were recruited at the Department of Public Health and Infectious Diseases of Sapienza University of Rome, Italy. The inclusion criteria were: (i) to have signed the informed consent, (ii) men at least 18 years of age, (iii) receiving cART, (iv) with HIV-1 RNA < 37 copies/ml and CD4^+^ T counts > 400 cells/mm^3^. Exclusion criteria were: (i) known or suspected allergy or intolerance to the specific probiotic formulation, (ii) use of probiotics or antibiotics during the 3 weeks prior to enrollment, (iii) drug addiction, (iv) history of or current inflammatory diseases of the small or large intestine, (v) diarrhea, (vi) any current, past or systemic malignancy. All patients collected fecal samples prior to initiation (T0) and after 6 months (T6) of probiotic supplementation.

Patients received 1.8 × 10^12^ live bacteria per day of the reference probiotic formulation (*L. plantarum* DSM24730, *S. thermophilus* DSM24731, *B. breve* DSM24732, *L. paracasei* DSM24733, *L. delbrueckii subsp. bulgaricus* DSM24734, *L. acidophilus* DSM 24735, *B. longum* DSM24736, *B. infantis* DSM24737). This formulation manufactured at Danisco/Dupont (USA) was previously commercialized under the brand VSL#3^®^, but now is available under the brand Vivomixx^®^ in Europe (Visbiome^®^ in USA, DeSimone Formulation in Korea). It was not possible to treat any patient with the VSL#3 manufactured at CSL/Nutrilinea, Italy, since preliminary data have shown that the CSL/Nutrilinea made formulation increases the levels of p24 (+8%), contrary to the Danisco-made product which has an inhibitory activity (4%), and this is obviously ethically inacceptable (8).

#### T-Cell Phenotyping by Flow Cytometry

Phenotypes and activation markers were evaluated by Miltenyi Biotec flow cytometer-MACSQuant Analyzer (8 fluorescence channels, three lasers) on freshly isolated peripheral blood mononuclear cells. Immune activation was evaluated by multiparameter flow cytofluorimetric analysis by the following anti-human monoclonal antibodies: CD3-PerCP, CD4-APC-Vio770, CD8-FITC, CD45RO-PE-Vio770, CD27-VioBlue, CD38-APC, and HLA-DR-PE (Miltenyi Biotec, Bergisch Gladbach, Germany).

#### Virological Analysis

HIV-1 RNA copy numbers were evaluated in plasma prepared from blood obtained in EDTA-containing tubes and stored at −80°C. Levels of HIV-1 RNA were measured with Versant kPCR (Siemens Healthcare Diagnostic Inc., Tarrytown, NY, USA) with a detection limit of 37 copies/ml.

#### Fecal Specimen Processing to Assess Patient Compliance

Bacterial DNA from patients’ fecal samples was extracted using the QIAamp DNA Stool Mini Kit (Qiagen, Hilden, Germany). Approximately 200 mg of feces were cut from frozen samples using a sterile disposable scalpel, resuspended in 1.4 ml of ASL lysis buffer from the stool kit, mixed with glass beads (150–212 µm Sigma-Aldrich, St. Louis, MO, USA), and homogenized thoroughly. The suspension was incubated at 95°C for 5 min and DNA was purified according to the manufacturer’s instructions. DNA was eluted in 200 µl of AE buffer (provided in the kit) and stored at −20°C.

#### Real-time PCR Assay

Real-time PCR was used to quantify bifidobacteria and lactobacilli using genus-specific primers and conditions described by Matsuki et al. (9) and by Stsepetova et al. (10), respectively. Briefly, PCR amplification and detection were performed on optical-grade 96-well plates using the Applied Biosystems 7500 Real-Time PCR instrument (Applied Biosystems Inc., Norwalk, CT, USA). To quantify bifidobacteria and lactobacilli, the reaction mixture (25 µl) was composed of SensiMix SYBR Low-ROX (BIOLINE, Taunton, MA, USA), 500 nM primers for *Bifidobacterium* genus and 200 nM for *Lactobacillus* genus and 2.5 µl of template DNA. The fluorescent products were detected at the last step of each of 40 cycles. A melting curve analysis was made after amplification to distinguish the targeted PCR product from the non-targeted PCR products. Standard curves were created using serial 10-fold dilutions of bacterial DNA extracted from *B. breve* and *Lactobacillus brevis*, respectively. All samples were analyzed in duplicate in two independent real-time PCR assays.

#### Fecal Metabolome

##### Sample Preparation

In order to study the fecal metabolome by NMR analysis, 80 mg of each fecal sample was vortex-mixed for 5 min with 1 ml of deionized water, followed by centrifugation for 15 min at 18,000 *g* at 4°C. 700 µl of the supernatant was added to 100 µl of a D_2_O solution of 3-(trimethylsilyl)-propionic-2,2,3,3-d4 acid sodium salt (TSP) 10 mM, set at pH 7.00 with 1 M phosphate buffer. Before analysis, the samples were again centrifuged.

##### ^1^H-NMR Spectra Acquisition

^1^H-NMR spectra were recorded at 298 K with an AVANCE III spectrometer (Bruker, Milan, Italy) operating at a frequency of 600.13 MHz. The water residual signal was suppressed by presaturation, while broad signals from slowly tumbling molecules were removed by including a CPMG (Carr-Purcell- Meiboom-Gill) filter to a free induction decay sequence. The filter was made up by a train of 400 echoes separated by 0.8 ms, for a total time of 328 ms. Each spectrum was acquired by summing up 256 transients using 32 K data points over a 7,211.54 Hz spectral (for an acquisition time of 2.27 s). In order to apply NMR as a quantitative technique (11), the recycle delay was set to 5 s, taking into consideration the longitudinal relaxation time of the protons under investigation.

#### Fecal Metabolome Data Analysis

The ^1^H-NMR spectra were adjusted for baseline irregularities as explained elsewhere (12). The signals were assigned by comparing their chemical shift and multiplicity with the Human Metabolome Database (13) and Chenomx software data bank (Chenomx Inc., Canada, version 8.1). Concentrations of molecules were calculated by employing the trimethylsilyl propionate (TSP) signal as an internal standard. In order to compensate for differences in dilution or solids content, all the spectra were normalized by means of probabilistic quotient normalization (14). The concentration of the molecules was expressed as millimoles per gram of fecal sample. Statistically significant differences were assessed by means of paired Wilcoxon–Mann–Whitney tests for paired samples (*p* < 0.05). Samples were considered as outliers, and therefore excluded, when their concentration at T0, at T6 or the T6 − T0 difference was outside 1.5 times the interquartile range (15).

### Comparative *In Vitro* Analysis of the Probiotic Formulations

Samples of the US-made “reference” formulation (lot TM091, expiry date expiry date 9/10/2017) and the Italian-made formulation (lot 512058, expiry date 12/2017) were utilized for the *in vitro* experiments. The bacterial strain content of the “reference” (US-made) formulation utilized in our trial is described in the *Participants and interventions*. The following strains: *L. plantarum* BP06, *S. thermophilus* BT01, *B. breve* BB02, *L. paracasei* BP07, *L. delbrueckii* subspecies *bulgaricus* BD08, *L. acidophilus* BA05, *B. longum* BL03, *B. infantis* BI04 are now instead present in the VSL#3^®^ commercialized in Europe and Canada and are produced at CSL, Italy. According to Ferring Pharmaceuticals, distributor of the product, the strains present in the Italy-made product are identical to the strains present in the US-made product. We did not utilize the Italy-made formulation in our clinical trials.

#### Culture Conditions, Bacterial Cell Counts, and Metabolome Analysis

The sachets containing the two formulations were handled according to the manufacturers’ instructions and were opened immediately before the assay. The sachet contents were suspended at 0.1% (w/v) in de Man, Rogosa and Sharpe (MRS) broth (Becton Dickinson and Company, Sparks, MD, USA), supplemented with 0.05% (w/v) l-cysteine. When indicated, inoculum was carried out in MRS broth 0.05% l-cysteine, added with 0.3 mmol/l or 0.6 mmol/l 1,3-dihydroxyacetone (DHA, Molbase Biotechnology, Shanghai, China). Bacterial suspensions were incubated at 37°C for 44 h in anaerobic jars supplemented with GazPack EZ (Becton Dickinson and Company). At the end of the incubation period, *Lactobacillus* and *Bifidobacterium* colony forming units (CFU) were determined by the plate count method on MRS agar plates; *S. thermophilus* CFU were determined analogously on M17 agar plates. The remaining cultures were centrifuged at 5,000 g for 10 min, then cell pellets were washed in sterile saline and resuspended in MRS medium supplemented with 10% glycerol, then frozen at −20°C; the supernatants were then filtered through a 0.2 µm membrane filter and stored at −20°C until NMR analysis. To study the metabolome of the probiotics, 1 ml of filtered supernatant was centrifuged for 15 min at 18,000 *g* and 4°C and then prepared for NMR analysis as described for the fecal metabolome. The molecular concentrations were expressed as millimoles per liter of culture medium.

### IEC-6 Cell Line and Culture Conditions

IEC-6 cell line (normal rat small intestine epithelial cells) provided by Sigma-Aldrich (St. Louis, MO, USA) were routinely monolayer-cultured in plastic culture flasks containing DMEM supplemented with 5% (v/v) fetal bovine serum, 0.1 IU/ml insulin, 2 mM l-glutamine, 100 U/ml penicillin, and 100 µg/ml streptomycin. After reaching 80% confluence, adherent cell cultures were expanded after previous detachment with trypsin solution from bovine pancreas. The well plates were incubated in sterile conditions at 37°C in a 5% CO_2_ humidified atmosphere and the complete medium was totally replaced every three days. For each experimental condition, IEC-6 cells were seeded within sterile 12- or 24-well plates (Becton Dickinson, San Jose, CA, USA), at 18,000 cells/cm^2^. At approximately 60% confluence, the cells were subjected to the different treatments. All culture reagents were acquired from Euroclone (Wetherby, West Yorkshire, UK). Cells were then incubated for 24 h with different DHA concentrations (0.1, 0.2, and 0.3 mmol/l) or with supernatants from bacterial suspensions grown for 44 h in presence or absence of 0.3 or 0.6 mmol/l DHA (final dilution 1:2).

#### IEC-6 Cell Viability Assay

After incubation, the cells were washed with phosphate-buffered saline (PBS), collected and centrifuged for 10 min at 400 *g*. Pellets were resuspended and incubated for 5 min with Trypan blue solution (0.04%, final concentration). Cells were counted in a Bürker chamber by optical microscopy (Eclipse 50i, Nikon Corporation, Japan). The cell numbers and the percentage of live and dead cells were registered. Non-treated cells were processed as negative controls.

#### Contrast Phase Microscopy

IEC6 cells were seeded on rounded coverslips coated with poly-l-lysine 0.01% (Sigma-Aldrich) in distilled water. Stocks of 1 g of each VSL#3 formulation (US-made reference version or Italian-made VSL#3) were suspended in 10 ml of PBS (Euroclone, Wetherby, West Yorkshire, UK) and added to IEC-6 cell line cultures at 1,000 bacterial cells/IEC-6 cell at 37°C. After 24 h, coverslips gently washed with PBS were first observed by contrast phase microscopy (Eclipse TS 100, Nikon Corporation, Japan) and then prepared for scanning electron microscopy (SEM) analysis, as described below.

#### Scanning Electron Microscopy Analysis

Stocks of 1 g of each VSL#3 formulation were suspended in 10 ml of PBS (Euroclone, Wetherby, West Yorkshire, UK). Bacteria were washed three times in PBS and then left to adhere for 1 h on rounded coverslips coated with Poly-l-Lysine 0.01% (Sigma-Aldrich) in distilled water; coverslips were fixed with 2% glutaraldehyde (Electron Microscopy Sciences, Hatfield, PA, USA) in PBS for 30 min, then dehydrated by subsequent exchanges of the following ethanol gradual series: 30, 50, 70, 90, and 100% ethanol diluted in distilled water. Samples were successively dried by evaporation of hexamethyldisilazane (HMDS; Electron Microscopy Sciences, Hatfield, PA, USA): samples were immersed in 100% HMDS for 3 min after the 100% ethanol step, then the excess of HMDS was removed by absorption on filter paper and desiccated for 25 min. Coverslips were glued onto stubs, coated with gold in a SCD040 Balzer Sputterer, and observed using a Philips 505 SEM at 10–20 kV.

IEC6 cells were prepared for scanning electron microscopy (SEM) analysis as follows: coverslips were fixed with 2% glutaraldehyde (Electron Microscopy Sciences, Hatfield, PA, USA) in PBS for 30 min, then dehydrated by subsequent exchanges of the following ethanol gradual series: 30, 50, 70, 90, and 100% ethanol diluted in distilled water. Samples were successively dried by evaporation of hexamethyldisilazane (HMDS; Electron Microscopy Sciences, Hatfield, PA, USA): samples were immersed in 100% HMDS for 3 min after the 100% ethanol step, then the excess of HMDS was removed by absorption on filter paper and desiccated for 25 min (16). Samples were glued onto stubs, coated with gold in a SCD040 Balzer Sputterer, and observed using a Philips 505 SEM at 20 kV.

#### *In Vitro* Monolayer Wound Healing Assay and Image Processing Methods

IEC-6 cells were cultured in 12-well microplates under normal culture conditions and allowed to proliferate until ~90% confluence was reached, then DMEM was removed from the well and cell monolayers were scratched using a 200 µl pipet tip to create a uniform cell-free wound area with reproducible width of wounding (0.7 mm). Debris was removed from the culture by gently washing with sterile PBS. Cell cultures were incubated for 24 h with fresh medium at 37°C in a 5% CO_2_ humidified atmosphere in the presence or absence of 0.3 mmol/l DHA. Non-treated cells were processed as negative controls.

Cell migration was monitored using an inverted microscope (Eclipse TS 100, Nikon) and photographed at different time intervals. At least three images for each condition were captured. To calculate the% closure of the wound, the images acquired for each sample (at least three measurements for each sample) were further analyzed quantitatively using the stand-alone TScratch software that was developed to automatically analyze wound healing assays (17).

#### Statistical Analysis

Statistical analysis was performed using Prism 6.01 GraphPad Software, San Diego, CA, USA. In order to assess the normality of the distribution, Shapiro–Wilk test was performed. When data were not normally distributed, logarithmic transformation was performed and the statistical significance was determined by paired two-tailed Student’s *t*-test. Changes were considered statistically significant if *p* values were <0.05.

## Results

### Participant Demographic and Clinical Characteristics

All study participants were HIV-1 positive Caucasian men. Median age was 41.8 years (IQR 22–53 years). They had been on cART for a median of 8 years (IQR 1.75–16.25 years). All subjects had been virologically suppressed (<37 HIV-1 RNA copies/ml) for at least 1 year and their median CD4^+^ cell count was 674 (IQR, 564–824 cells/mm^3^) and 683 (IQR, 610–818 cells/mm^3^) cells/mm^3^ before and after supplementation with the reference formulation.

### Participant Compliance with Probiotic Supplementation

**Figure 1:**
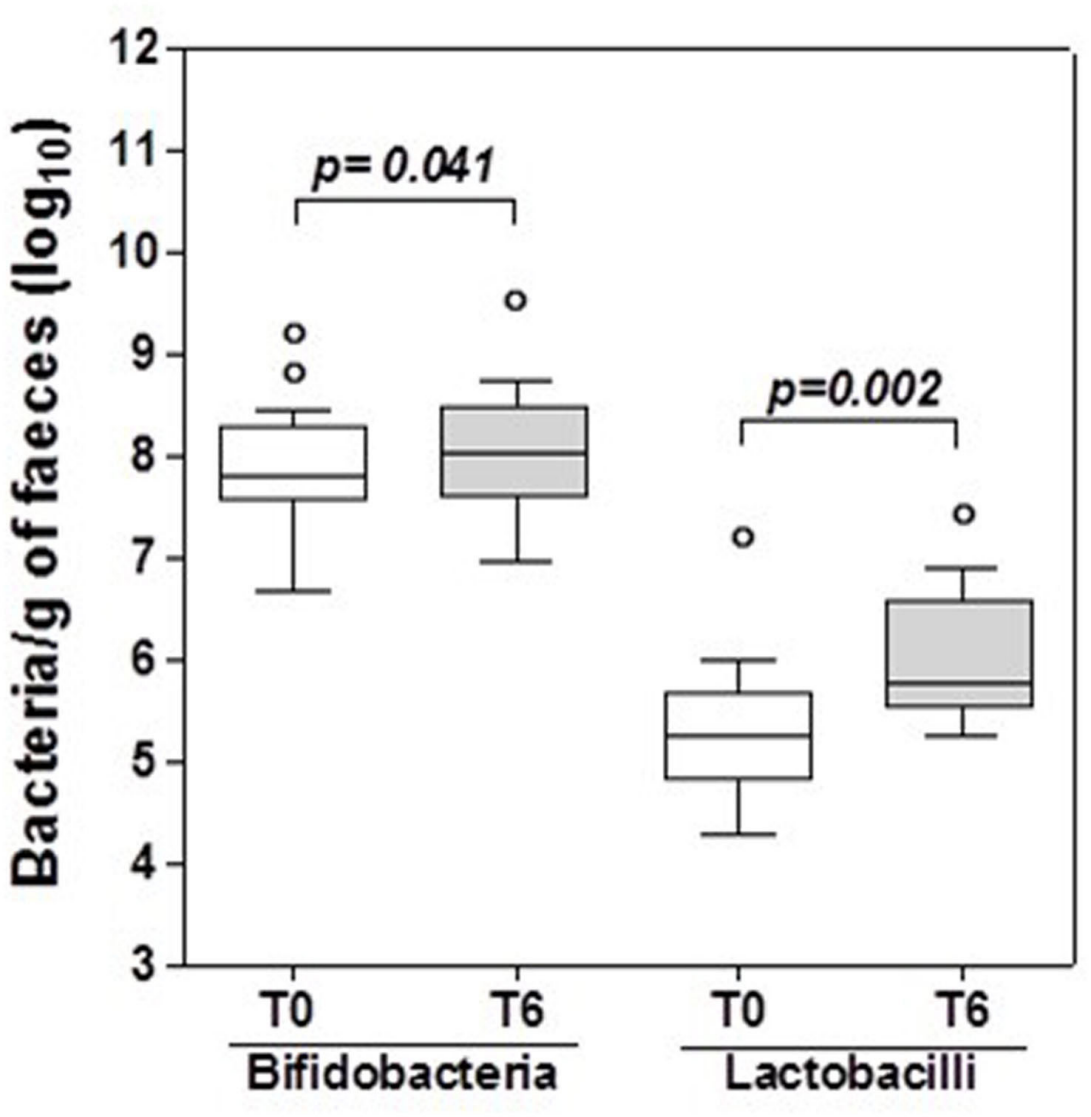
Bifidobacteria and lactobacilli in HIV-1 positive patients’ stools. Box and whisker plots based on log10 16S rRNA gene copies per gram stool. The horizontal line in the middle of each box represents the median, while the top and bottom borders represent the 75th and 25th percentiles, respectively. The outliers are represented as individual points outside the boxes. Statistical tests were performed using paired *t*-test.

The number of bifidobacteria and lactobacilli in fecal samples was assessed at enrollment (T0) and after 6 months of probiotic supplementation (T6). At T6 there was a significant increase in the specific bacterial genera contained in the probiotic supplement compared with T0 (*p* < 0.05 for both), and this was assumed as proof that the participants had complied with the probiotic supplementation (Figure 1).

### Changes in T-Cell Activation in Peripheral Blood after 6 Months of Probiotic Supplementation

T-cell activation markers HLA-DR^+^ and CD38^+^ (CD38^+^HLA-DR^+^) were analyzed in CD4^+^ T-cell subsets from peripheral blood of the HIV-1 infected participants before and after supplementation with the US-made probiotic. The frequencies of CD4^+^ cells expressing simultaneously expressing HLA-DR and *CD38* significantly decreased after 6 months of supplementation with the US-made probiotic {CD4^+^ T-cells: [median 0.22 (IQR: 0.10–0.39)] compared to T0 [median 0.63 (IQR: 0.35–2.09)]; *p* < 0.005}.

### Virological Analysis

HIV-1 RNA copy numbers after 6 months of probiotic supplementation were confirmed to be persistently under 37 copies/ml, thereby excluding the possibility that the reference formulation probiotic has any facilitatory role for the replication of HIV in cART patients.

#### Probiotic Supplementation Reduces the Fecal Concentration of Six Molecules

Untargeted metabolomic analysis of the participants’ feces by ^1^H-NMR led to the identification of 59 resolved signals pertaining to different molecules with an intensity above the limit of quantification. Fifty-four were assigned mainly to amino acids and their derivatives, short-chain fatty acids, organic acids, and monomeric carbohydrates, while five could not be assigned. A paired comparison revealed that the concentrations of 10 molecules were affected by the treatment, namely tryptophan, phenylalanine, tyramine, tyrosine, p-cresol, arabinose, 1,3-dihydroxyacetone, glycine, dimethylamine (DMA), and pyruvate. All molecules but tyrosine and p-cresol showed a decrease between T0 and T6, as detailed in Table 1.

**Table 1:** Fecal metabolites affected by probiotic treatment.

Metabolite	T0	T6
Tryptophan	9.48E−5 ± 6.86E−5	5.26E−5 ± 7.79E−5
Phenylalanine	3.04E−3 ± 3.21E−3	1.86E−3 ± 1.14E−3
Tyramine	1.83E−3 ± 9.50E−4	1.52E−3 ± 8.26E−4
Tyrosine	2.22E−4 ± 1.24E−4	4.53E−4 ± 4.23E−4
p-Cresol	3.55E−4 ± 2.35E−4	3.57E−4 ± 1.46E−4
Arabinose	1.50E−4 ± 2.22E−4	9.40E−5 ± 9.65E−5
1,3-Dihydroxyacetone	5.89E−4 ± 3.08E−4	4.74E−4 ± 3.67E−4
Glycine	3.99E−3 ± 2.37E−3	3.70E−3 ± 3.47E−3
Dimethylamine	4.44E−4 ± 1.97E−4	3.69E−4 ± 1.98E−4
Pyruvate	3.05E−4 ± 1.70E−4	2.92E−4 ± 3.58E−4

*Concentration of the molecules identified by 1H-NMR in the feces of subjects before (T0) and after 6 months of treatment (T6) with probiotics (millimoles per gram of feces). For clarity, only the molecules whose concentration was statistically affected (*p* < 0.05) by the treatment are reported*.

#### The US-Made and Italian-Made Formulations Have a Different Capability to Produce and Metabolize DHA, which Affects *S. thermophilus* Viability

Each probiotic mix was cultivated up to the stationary phase of growth (44 h), then the metabolomic analysis was done on the bacterial supernatant, looking for the same metabolites (tryptophan, phenylalanine, tyramine, tyrosine, p-cresol, arabinose, 1,3-dihydroxyacetone, glycine, DMA, and pyruvate) that were found to be modified in the feces of participants after probiotic supplementation (Table 2).

**Table 2:** Concentration of metabolites in VSL#3 culture supernatants.

	MRS	US-made	Italian-made
Tryptophan	9.26 × 10^−1^	8.19 × 10^−1^	8.71 × 10^−1^
Phenylalanine	4.26	4.51	4.21
Tyramine	N.D.	N.D.	N.D.
Tyrosine	1.82	2.30	2.13
p-Cresol	N.D.	N.D.	N.D.
Arabinose	N.D.	N.D.	N.D.
1,3-Dihydroxyacetone	5.98 × 10^−2^	1.71 × 10^−2^	2.40 × 10^−1^
Glycine	N.D.	N.D.	N.D.
Dimethylamine	N.D.	N.D.	N.D.
Pyruvate	1.83 × 10^−1^	2.89 × 10^−1^	3.03 × 10^−1^

*Concentration of the molecules identified by 1H-NMR in the VSL#3 supernatants after incubation in MRS medium for 44 h (millimoles per liter). Only the molecules found to be modified in the patients after the probiotic treatment are reported. In addition, the concentrations of these molecules in MRS prior to VSL#3 addition are reported*.

Tyramine, p-cresol, arabinose, glycine, and DMA could not be identified in the studied samples. Both probiotic formulations showed similar concentrations of tryptophan, phenylalanine, tyrosine, and pyruvate at the end of the incubation period. While the US-made “reference” formulation reduced the amount of DHA in the medium, the Italian-made formulation increased the amount of DHA detectable in the medium (Table 2).

To investigate the effects of DHA levels on the probiotic bacteria growth and metabolism, we repeated the experiments by adding exogenous DHA at two different concentrations, one comparable to the amount of DHA previously detected in the Italian-made supernatant (0.3 mmol/l) and the other corresponding to a doubled amount. The results confirmed that the US-made formulation was able to metabolize the DHA present in the culture medium, reducing its concentration by up to 10^−2^ mmol/l in any experimental conditions; on the contrary, the Italian-made cultures were characterized by higher levels of DHA, significantly above the background of culture medium (Table 3). Furthermore, when DHA was added to the bacterial cultures at the concentration of 0.6 mmol/l, it significantly affected the viability of *S. thermophilus* present in the formulation, suggesting that any dietary intervention (probiotics, prebiotics, fermented foods, etc.) able to modify the host’s fecal metabolic profile in the direction of an increased DHA level, may impact on the growth and/or viability of certain bacterial populations, as previously reported for *E. coli* (Table 4).

**Table 3:** Concentration of 1,3-dihydroxyacetone (DHA) in US-made and Italian-made probiotics culture supernatants.

	Medium	US-made VSL#3	Italian-made VSL#3
MRS + 0.3 mmol/l DHA	3.45 × 10^−1^	2.22 × 10^−2^	2.59 × 10^−1^
MRS + 0.6 mmol/l DHA	6.45 × 10^−1^	4.10 × 10^−2^	5.19 × 10^−1^

*Concentration of DHA (millimoles per liter) in culture media MRS (4.46 × 10^−2^ mmol/l) added with 0.3 mmol/l or 0.6 mmol/l DHA after 44 h of incubation with US-made and Italian-made VSL#3*.

**Table 4:** Effect of 1,3-dihydroxyacetone (DHA) on VSL#3 bacterial mixture viability.

	US-made VSL#3		Italian-made VSL#3	
	
	*Lactobacillus*/*Bifidobacterium*	*S. thermophilus*	*Lactobacillus*/*Bifidobacterium*	*S. thermophilus*
MRS	8.38 × 10^8^ ± 1.49 × 10^8^	1.01 × 10^9^ ± 4.15 × 10^8^	7.83 × 10^8^ ± 2.02 × 10^8^	1.08 × 10^9^ ± 2.41 × 10^8^
MRS + 0.3 mmol/l DHA	6.7 × 10^8^ ± 2.22 × 10^8^	8.1 × 10^8^ ± 4.04 × 10^8^	1.17 × 10^9^ ± 4.74 × 10^8^	9.08 × 10^8^ ± 9.06 × 10^8^
MRS + 0.6 mmol/l DHA	9.38 × 10^8^ ± 6.52 × 10^8^	5.9 × 10^8^ ± 2.33 × 10^8^	8.1 × 10^8^ ± 6.7 × 10^8^	6.5 × 10^8^ ± 2.15 × 10^8^*

*Lactobacillus/Bifidobacterium, and S. thermophilus bacterial counts (CFU/ml) after 44 h of culture. US-made and Italian-made probiotic mixes were grown in MRS and MRS added with 0.3 mmol/l of 0.6 mmol/l DHA. Data are expressed as mean ± SD*.

*p-Values < 0.05 (calculated by Student’s t-test).

#### Different Effects of the Two Formulations on IEC-6 Cells

**Figure 2:**
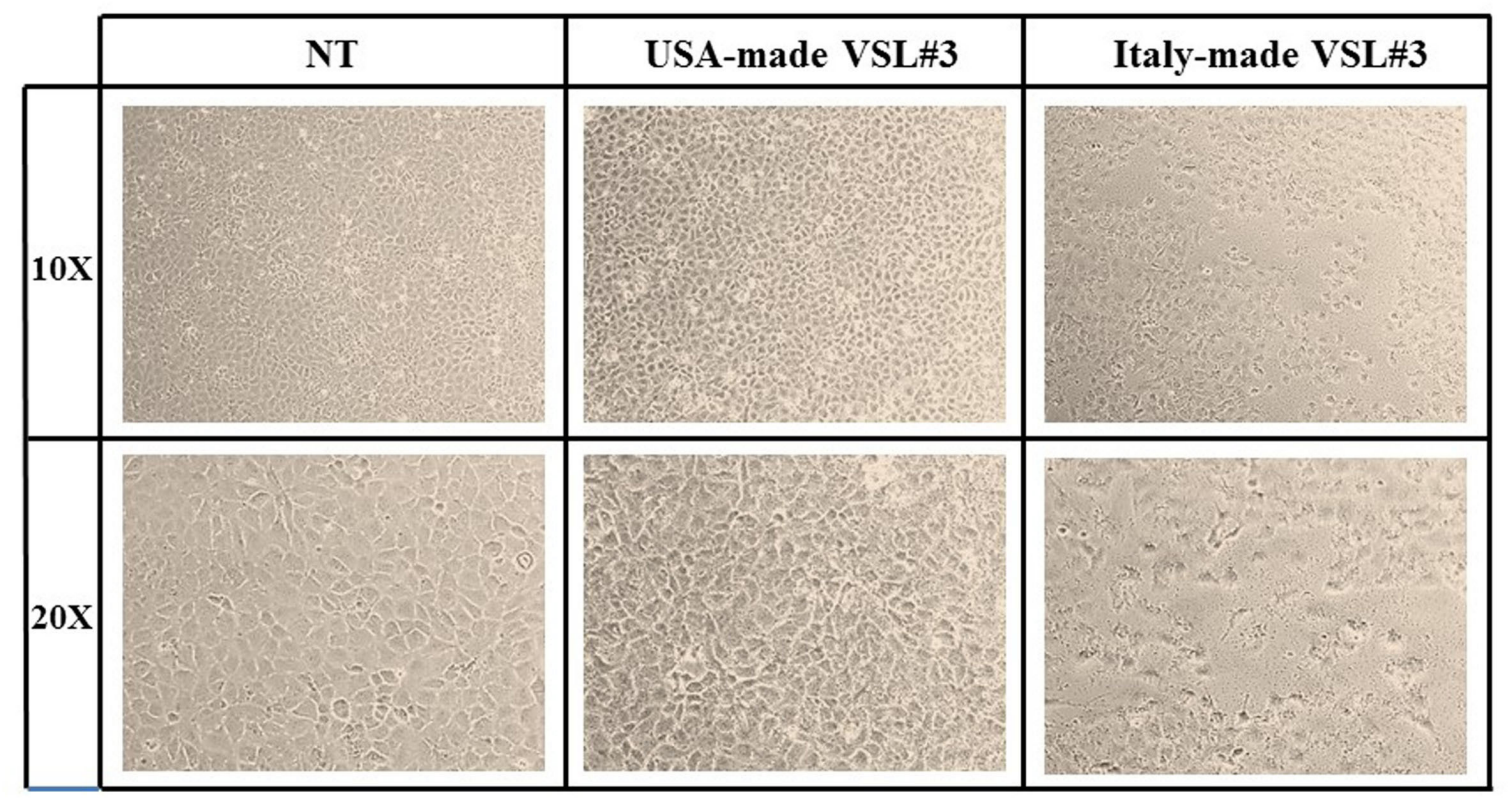
Effects *in vitro* of USA-made VSL#3 bacteria on IEC-6 cell cultures. IEC-6 cultures were mixed with bacterial suspensions prepared from US- and Italian-made VSL#3 at 1,000 bacterial cells/IEC-6 cell to highlight possible effects on morphology and cellularity after 24 h incubation at 37°C. The phase contrast microscopy images shown are representative of two independent experiments (magnification 10× or 20×).

**Figure 3:**
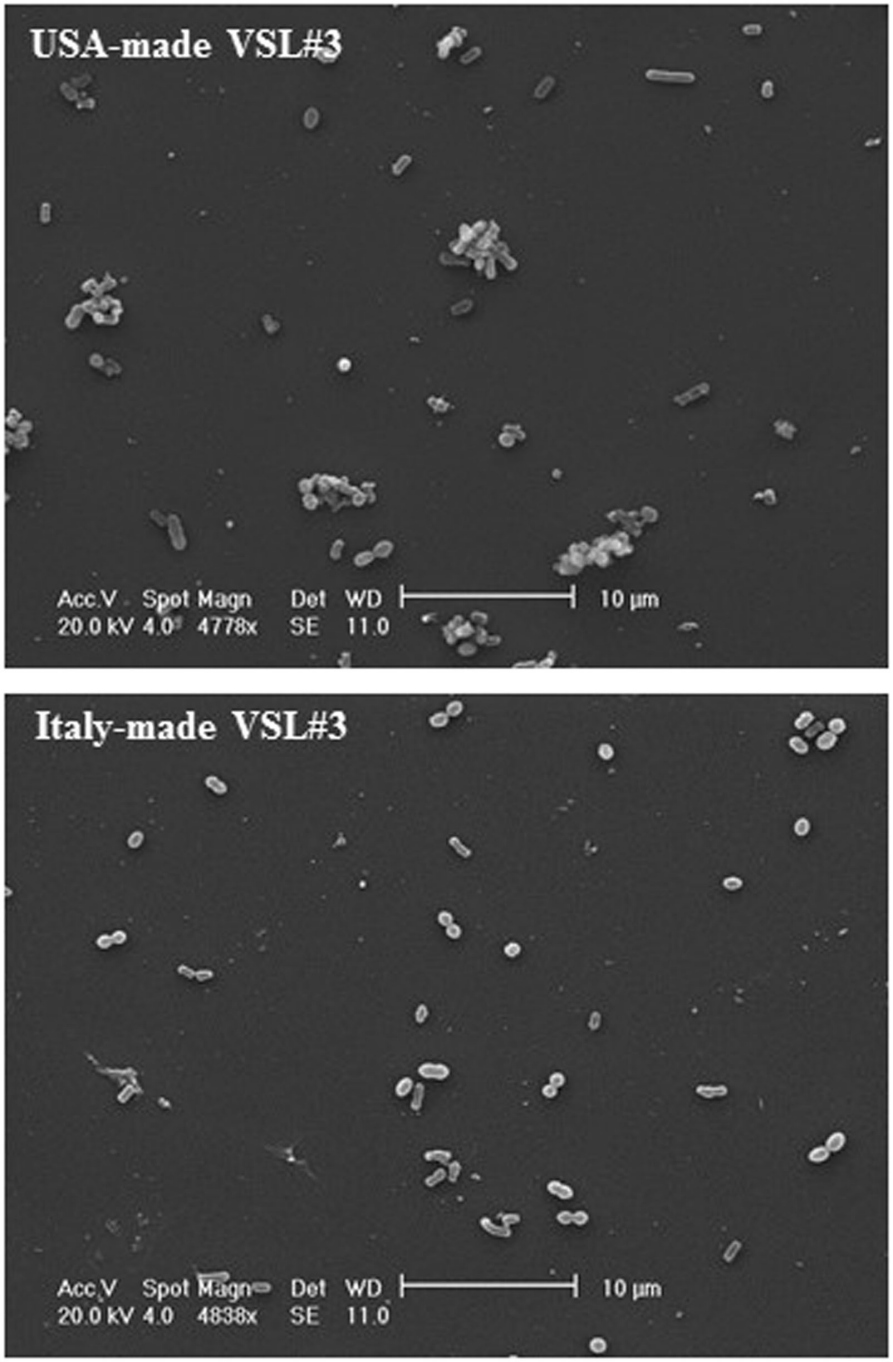
Scanning electron microscopic analysis of bacteria from USA-made VSL#3. The images shown at different magnifications are representative of two independent analyses.

**Figure 4:**
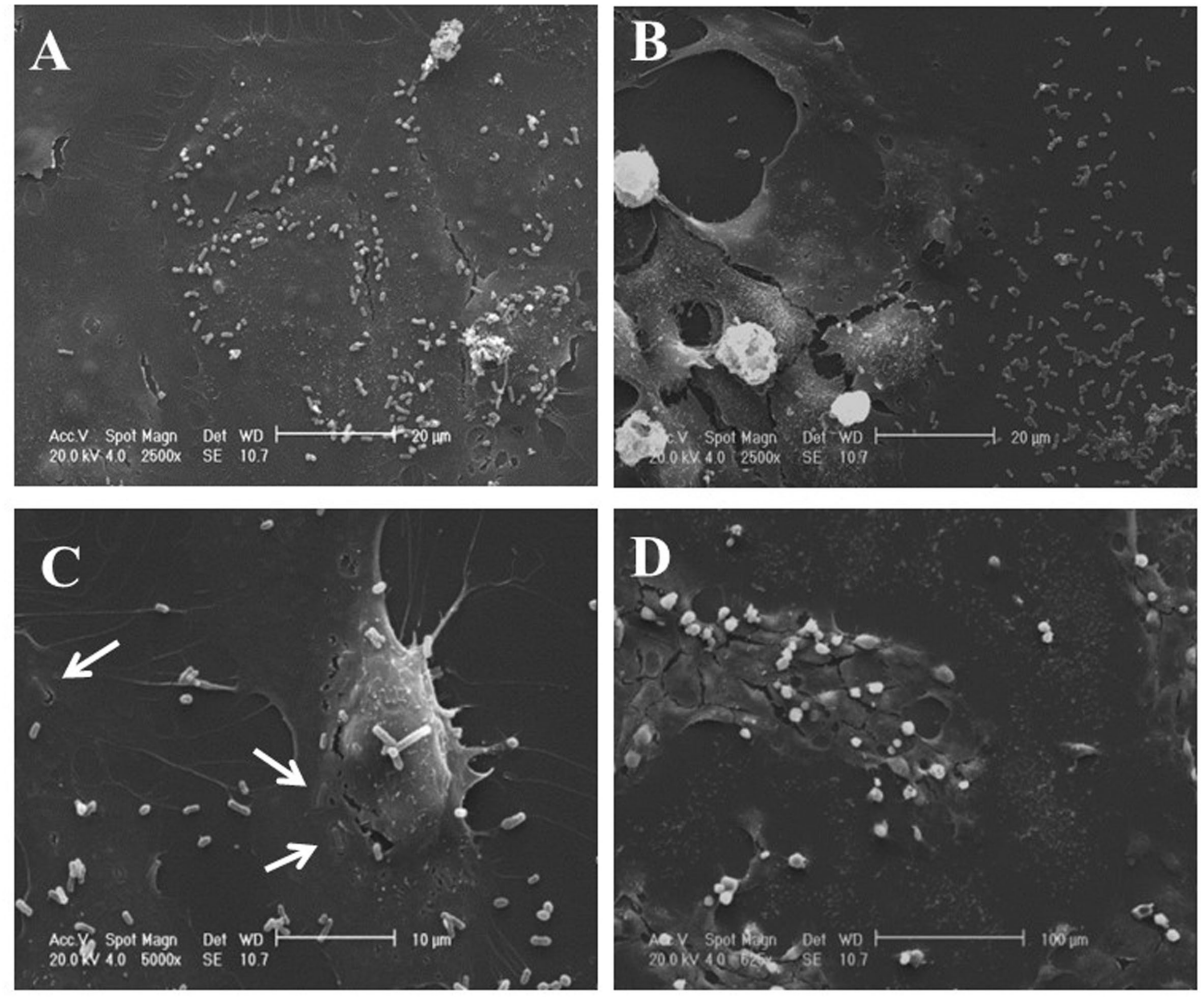
Scanning electron microscopic analysis of IEC-6 cell cultures treated with USA-made VSL#3 bacterial suspension. IEC6 cells were seeded on rounded coverslips coated with Poly-l-Lysine. US- or Italian-made VSL#3 resuspended in phosphate-buffered saline were added to IEC-6 cell line cultures at 1,000 bacterial cells/IEC-6 cell at 37°C for 24 h. The images shown are representative of two independent experiments. **(A,C)** Representative images of IEC-6 treated with US-made bacteria. **(B,D)** Representative images of IEC-6 treated with Italian-made bacteria. Arrows indicate the integration process of bacteria from US-product on cell surface which was not observed with bacteria from the Italian-made formulation.

IEC-6 cells were mixed with bacterial suspensions prepared from the two probiotic formulations at 1,000 bacterial cells/IEC-6 cell to highlight possible effects on morphology and cellularity. As evidenced by the phase contrast microscopic images shown in Figure 2, while the bacteria from US-made (reference) VSL#3 did not affect morphology and cellular density of the monolayer, the addition of bacteria from Italian-made VSL#3 caused clear morphological cell damage and strongly reduced cellularity of the IEC-6 monolayer, thus confirming previously published data (18). SEM analysis of bacteria did not show significant differences between the formulations, although there was a greater tendency to aggregation of the bacteria from the US-made sachet, while bacteria from the Italian-made VSL#3 appeared mostly isolated (Figure 3). SEM observation of IEC-6 incubated for 24 h in the presence of VSL#3 showed that US-made bacteria were largely attached to cells (Figure 4A) with frequent cases of internalization (Figure 4C). However, the Italian-made bacteria appeared mostly away from cells, with rare cases of membrane adhesion and internalization (Figures 4B,D).

**Figure 5:**
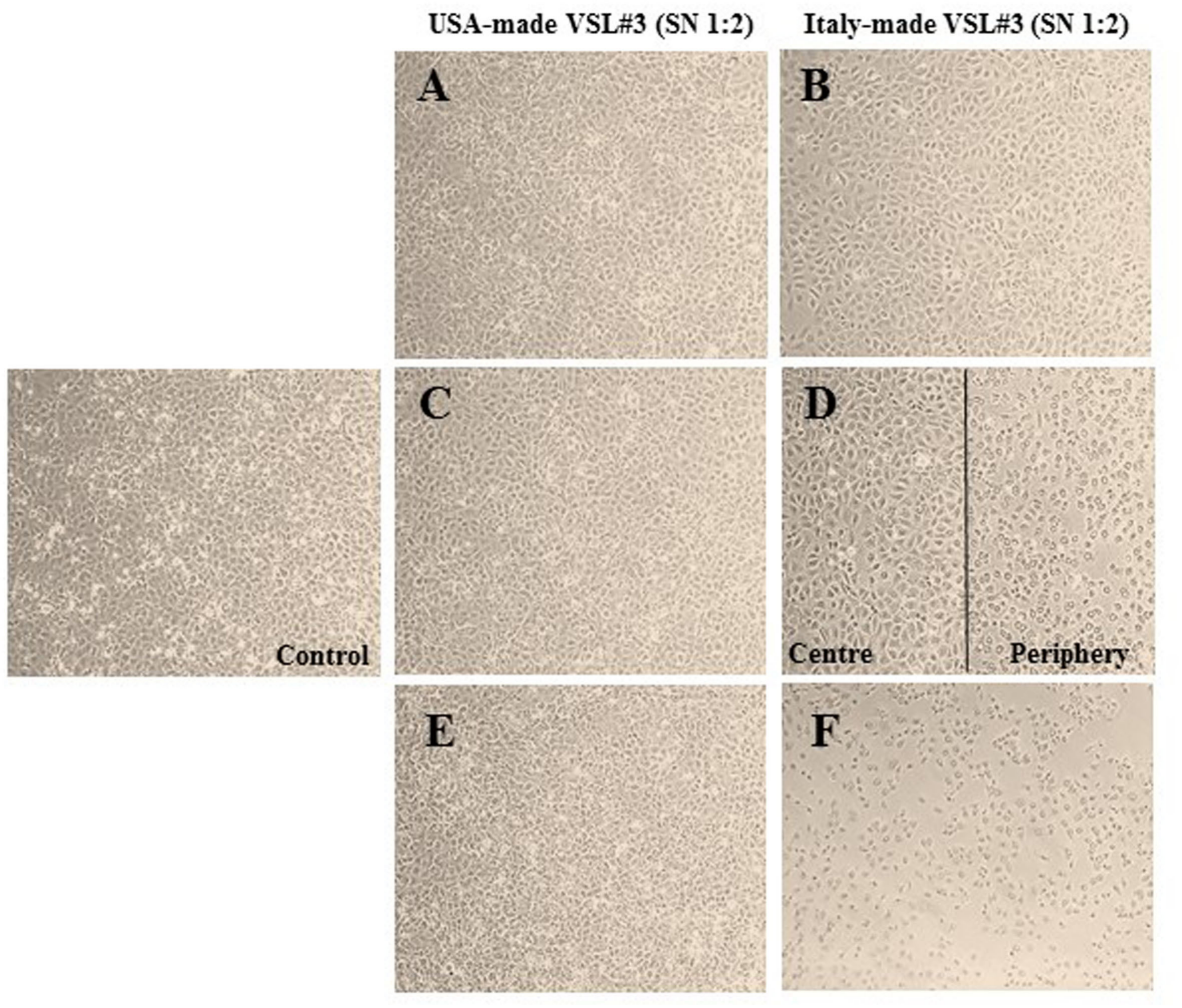
Effects *in vitro* of conditioned medium from USA-made VSL#3 bacteria grown for 44 h in presence or absence of 0.3 or 0.6 mmol/l 1,3-dihydroxyacetone (DHA) on IEC-6 cell cultures for 24 h (final dilution of conditioned medium on cell cultures: 1:2). **(A,B)** Cells treated with conditioned medium from US- or Italian-made VSL#, respectively. **(C,D)** Cells treated with conditioned medium from US- and Italian-made VSL#3 grown with 0.3 mmol/l DHA, respectively. **(E,F)** Cells treated with conditioned medium from US- and Italian-made VSL#3 grown with 0.6 mmol/l DHA, respectively. An image of untreated cells (Control) is also shown. The phase contrast microscopy images shown (10× magnification) are representative of two independent experiments.

Based on the results described above of the metabolomic analysis of the two formulations sold under the brand VSL#3, we also analyzed the effects of conditioned MRS medium from US- or Italian-made bacteria cultured for 44 h in the presence or absence of DHA at 0.3 or 0.6 mmol/l on IEC-6 cells after 24 h incubation. Addition of conditioned MRS medium to the cell cultures at 1:2 dilution meant that original DHA concentrations (0.3 or 0.6 mmol/l) were also diluted to obtain a final concentration of 0.15 or 0.3 mmol/l, respectively. As shown in Figure 5, while no evident effects could be observed in the cells incubated with conditioned MRS medium from US-made bacteria cultured in the absence (Figure 5A) or presence of DHA (Figures 5C,E), significant IEC-6 cell damage was evident in the samples treated with Italian-made bacteria cultured in the presence of DHA as compared to conditioned MRS medium without DHA addition (Figure 5B). In particular, in the cell cultures where the final virtual concentration of DHA was 0.15 mmol/l, the toxic effect was mainly evident on the periphery of wells (Figure 5D), while dramatic cell damage was extended to the whole wells where the final virtual concentration of DHA was 0.3 mmol/l (Figure 5F).

**Figure 6:**
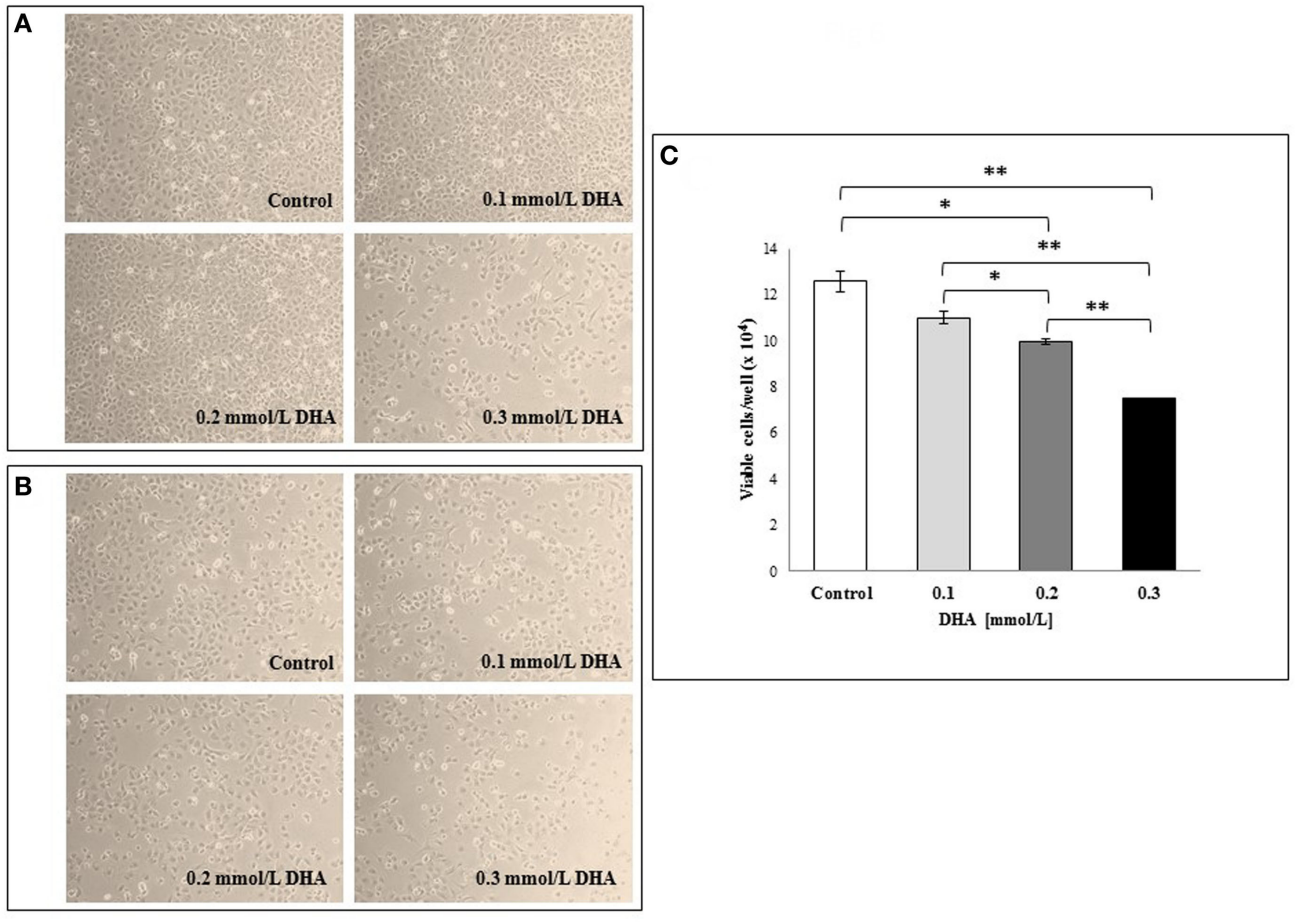
Effects *in vitro* of 1,3-dihydroxyacetone (DHA) on IEC-6 cell cultures. IEC-6 cells were incubated with different concentrations of DHA (0.1, 0.2, and 0.3 mmol/l) for 24 h incubation at 37°C. Phase contrast microscopy representative images of the cells at the center of the well **(A)** and at the periphery **(B)** are shown (10× magnification). **(C)** Effect of DHA on cell viability assessed by Trypan blue dye exclusion. The results are expressed as mean values of duplicates ± SD. *p < 0.05; **p < 0.01; ***p < 0.001. The results are representative of two independent experiments.

Based on these findings, we wanted to verify the potential *in vitro* toxicity of DHA at different concentrations (0.1, 0.2, and 0.3 mmol/l) on IEC-6 cells after 24 h incubation. The representative light microscopy images captured in both the center and periphery of plate wells (Figures 6A,B, respectively), showed DHA concentration-dependent cell damage and decrease in cell numbers that was more evident on the well periphery. The images from untreated cells (control) are also shown. In Figure 6C, the results of a cell viability assay with Trypan blue dye exclusion test expressed as the mean of duplicate values ± SD are shown. As expected, a dose-dependent decrease in viable IEC-6 cell number compared to control was evident at all DHA concentrations after 24 h incubation. The inhibitory effect of DHA on IEC-6 cell growth, which was evident, even if not statistically significant, at 0.1 mmol/l, appeared significantly different at higher concentrations (0.2 and 0.3 mmol/l) when compared to untreated cells (*p* < 0.05 and *p* < 0.01, respectively).

**Figure 7:**
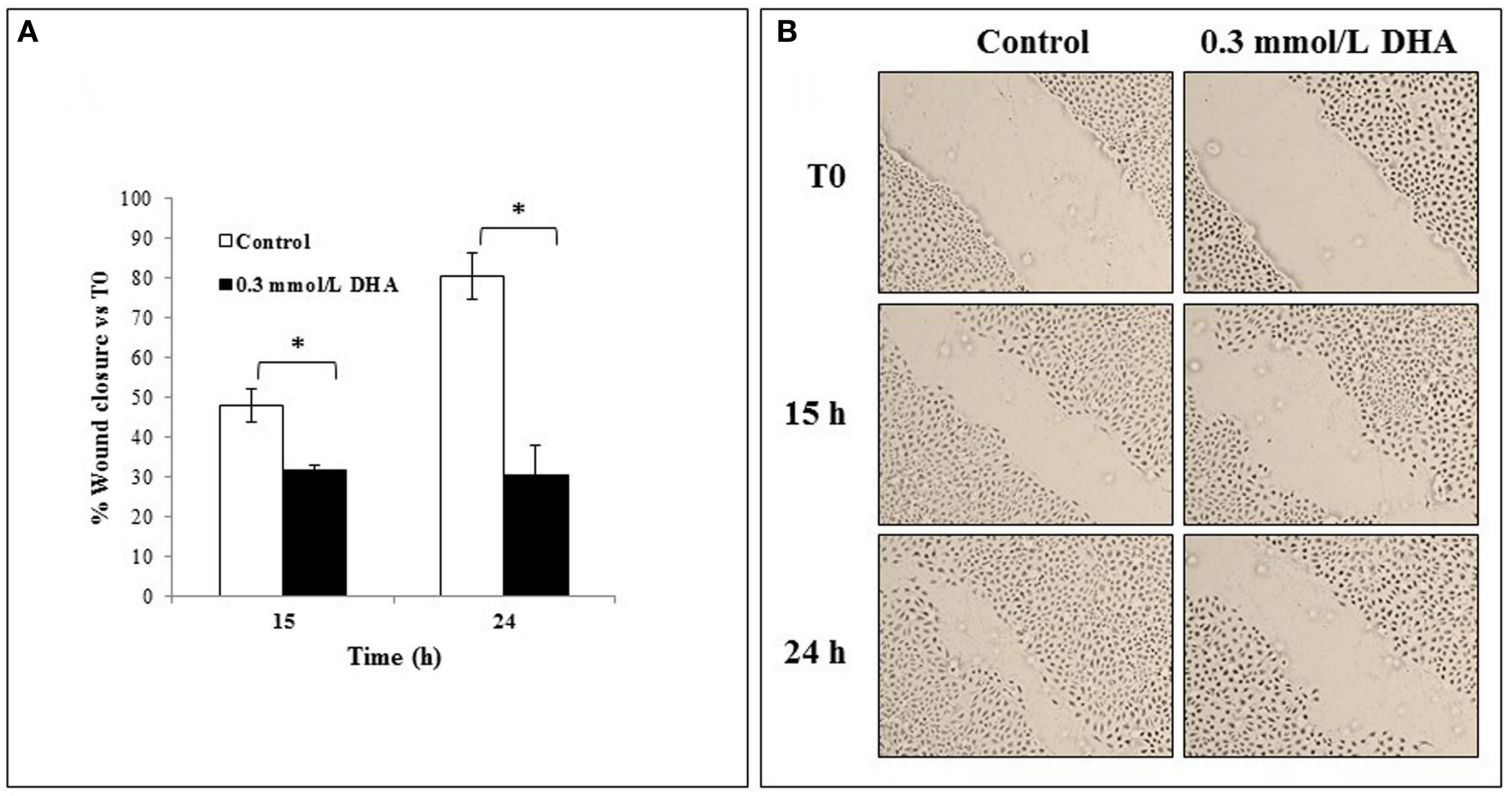
Effects of 1,3-dihydroxyacetone (DHA) on scratched monolayer wound healing of IEC-6. **(A)** The effect of DHA (0.3 mmol/l) on the closure rate of scratched monolayers was analyzed and compared to control cells. The microscopy images acquired for each sample were analyzed with TScratch software to automatically quantify the percentage of wound closure. Data are presented as the mean of duplicates ± SD and are relative to% wound closure versus T0 at 15 and 24 h from monolayer scratching, as indicated. The results are representative of two independent experiments (*p < 0.05; **p < 0.01). **(B)** Representative microscopy images (10× magnification) of all cell culture conditions are shown.

The effect of 0.3 mmol/l DHA on the rate of scratched monolayer closure was also analyzed and compared to the relative untreated cells at 0 h, as described in the Section “Materials and Methods.” The percentages of wound closure in untreated and DHA-treated cells were evaluated by observing the re-population of the area between the wound edges at different time points after the lesion (15 and 24 h). As shown in Figure 7A, the treatment with DHA led to a lower rate of monolayer repair with respect to untreated control, and this was statistically significant both after 15 and 24 h (*p* < 0.05). Representative images from microscopic observations of scratched monolayers untreated or treated with DHA are shown in Figure 7B.

## Discussion

Human immunodeficiency virus infection is characterized by a severe dysbiosis with a depletion of some beneficial bacteria, i.e., lactobacilli and bifidobacterial (19–21). Consequently, it has been suggested that the restoration of such species could somehow contribute to the recovery of an adequate mucosal immune response and potentiate the antiviral defenses. In fact, it has recently been shown that vaginal lactobacilli are able to inhibit HIV-1 replication in human tissues *ex vivo* (22), and counteract infections by other sexually transmitted pathogens (23–26). In animal models, probiotics had a beneficial impact on mucosal health, enhancing local cellular and humoral immune defenses against HIV (27, 28). d’Ettorre et al. have recently shown that supplementing cART with the probiotic formulation described in the current paper (the “reference” formulation) reduces systemic immune activation, and improves gut immune restoration and brain function (4).

However, not all probiotic formulations are effective and recommended for HIV patients. In a placebo-controlled clinical study, cART patients taking a four-strain probiotic product for 2 weeks failed to reduce their gastrointestinal symptoms notwithstanding increased gut levels of *Lactobacillus* after the treatment (29). Haghighat and Crum-Cianflone identified 10 cases among HIV patients who developed lactobacillemia after probiotic supplementation, suggesting the low count of CD4 T cells (<50 cells/mm^3^) and altered integrity of the mucosal barrier as potential risks for systemic infection (30). Among the possible hypotheses for why some probiotic formulations are effective and others are ineffective, the role played by the manufacturing of the probiotic is rarely taken into consideration. The dogma that genetic identity results in identical safety and efficacy has been strongly challenged by Sanders et al. (1), who wrote that “*growth conditions, growth substrates, cryoprotectants, food formulation, food processing conditions, and storage conditions may affect probiotic properties as scientists seek to optimize processes, viability, and function. Such modifications concomitantly may generate detectable differences in genes (mutations, genome rearrangements), gene expression patterns, or metabolic output. This raises the question of when such changes warrant a re-examination of efficacy or safety*.”

Even though, according to Ferring, the distributor of the VSL#3^®^, the strains present in the Italy-made product are identical to the strains present in the US-made product, recently, Cinque et al. have shown that the VSL#3 formulations manufactured in the US and Italy have a different effect on tumor cell lines and wound healing (18, 31). The same discrepancies between VSL#3 formulations manufactured at different sites have been reported by Biagioli et al. in animal models of IBDs (32). These discrepancies may have a major impact on patient safety and on the liability of doctors when they prescribe a probiotic formulation made with different processes at different production sites from the formulation, which generated the original evidence, without properly informing the patients.

In cART patients, a number of clinical studies conducted with the “reference formulation” (2–4) have reported beneficial effects at the intestinal and neurological levels, confirming and extending what previously observed in SIV-infected monkeys (5–7). This formulation is now no longer available in Europe, Canada, and some other countries under the VSL#3^®^ brand, which instead is now applied to a different probiotic mix that is compared with the “reference formulation” in the work described here. The fact that the new formulation is now dairy-free means that the growth media for the bacterial strains have been changed, and the fact that the manufacturing has been moved from the US (Danisco/Dupont) to Italy (CSL/Nutrilinea) may well have given rise to genome rearrangements, different gene expression patterns, or divergent metabolic output (1).

The data here reported confirm previously published reports of a reduction in immunoactivation after 6 months of treatment with the “reference formulation,” with a statistically significant reduction in the percentage of CD4^+^CD38^+^HLA-DR^+^ T-cells at 6 months [median 0.22 (IQR: 0.10–0.39)] compared to baseline [median 0.63 (IQR: 0.35–2.09)]. These findings reinforce the concept on the beneficial impact of probiotic intervention with the US-made “reference formulation” on systemic immune activation in chronically HIV-1 infected patients. The viral load was persistently below 37 copies/ml, and this is an additional indicator of the formulation’s safety. No adverse events were registered, and notably at the end of the trial all the subjects expressed the desire to continue dietary supplementation with the same probiotic preparation because they experienced a subjective improvement of wellness, as previously documented (4).

The ability of the US-made (“reference formulation”) probiotic to restore lactobacilli and bifidobacteria was confirmed by a significant (*p* < 0.05) increase in these specific genera in the fecal samples of the patients at 6 months compared to baseline (Figure 1). Since the probiotic formulation administered to our patients contains metabolically active bacteria, we assumed that patients’ fecal metabolomic profile would have been changed at the end of the 6 months of treatment. Ascertained these modifications in the feces, the next step has been the evaluation of both formulations for their capacity to produce or metabolize the same molecules *in vitro*, and their biological effects.

Metabolomic analysis of the feces was then performed, on the assumption that being downstream of the genome, transcriptome, and proteome, the metabolome would be the best representation of the microbiota phenotype, and that changes in individual metabolites might be significant even in case of small changes on metabolic fluxes, due to cascade or feedback effects. Out of 54 assigned molecules, mainly pertaining to the classes of amino acids, organic acids and carbohydrates identified in the feces of the patients, we focused our attention on tryptophan, phenylalanine, tyramine, tyrosine, p-cresol, arabinose, 1,3-dihydroxyacetone, glycine, DMA, and pyruvate, which were significantly affected by the 6 months of probiotic supplementation (Table 1). Special attention was paid to DHA, a triose sugar generated from fructose-1-phosphate and fructose-1,6-diphosphate catalyzed by aldolase B activity (33). The reduction in DHA in the feces after the probiotic treatment was considered one of the signs of an amelioration of the patients’ gut microbiota, in light of the fact that DHA has been shown to be mutagenic in the *Salmonella* mutagenicity assay and an inducer of DNA damage (34, 35). When DHA accumulates, it indicates an alteration of the glycolytic pathways, and it tends to react with proteins by the Maillard-type reaction, inducing DNA damage, apoptosis, and cell-cycle block (36). Further studies were also carried out on other molecules of interest, and will be published separately.

The next step was aimed at assessing whether both probiotic formulations may produce or metabolize DHA when cultivated up to the stationary phase of growth (44 h), and the influence of DHA on the bacterial cells during the fermentation when it is exogenously added. Our *in vitro* experiments confirm the capability of the US-made VSL#3 to metabolize DHA, as reflected by a reduced concentration of this metabolite in the culture medium (Table 2), confirming what was observed in feces from cART patients after a 6 months’ treatment. This metabolic feature was not evidenced for the Italian-made product, which on the contrary produced significantly higher levels of DHA. Interestingly, when DHA was added to the bacterial cell cultures, a significant (*p* < 0.05) decrease in *S. thermophilus* viability was observed and this may alter the biochemical and immunological characteristics of the formulation (Table 4). Previously, Cinque et al. have shown that the Italian-made VSL#3 contains approximately 130% more dead bacteria than the US-made VSL#3 (31). According to our data, one of the causes for the reduced viability of the bacterial cells present in the Italian-made product is the presence of strains which synthesize DHA, depending on gene expression in different culture media and the production processes of the strains at CSL, and which antagonize the survival of other bacterial species, in the specific case *S. thermophilus*.

To clarify what the impact would be of probiotic supplementation that increases DHA levels in the gut, we decided to add the supernatant from the two probiotic products to IEC-6 cells. While the US-made VSL#3 did not affect the morphology and cellular density of the IEC-6 monolayer, the Italian-made product strongly reduced the viability of the IEC-6 monolayer, thus reconfirming previously published data (18). SEM analysis did not evidence significant differences between US- or Italian-made VSL#3, although there was a greater tendency to aggregation in bacteria from the US-made formulation. Moreover, SEM also showed that the US-made bacteria showed greater ability to adhere to IEC-6 cells. Considering that the non-specific binding of probiotic bacteria to cell wall constituents has been recently hypothesized as a potential mechanism underlying the ability of probiotics to block the accessibility of pathogen agents (37), the differences we have observed between the two bacterial formulations, although they require further investigation, appear intriguing.

Finally, in an attempt to link the presence of certain levels of DHA to the observed cellular damage, conditioned MRS medium from US- or Italian-made bacteria cultured in the presence or absence of DHA was added to the IEC-6 cells. DHA dose-dependent cell damage was observed in the cultures incubated with Italian-made VSL#3, while no effects could be observed with conditioned MRS medium from the US-made product cultured in the absence or presence of DHA. The DHA concentration-dependent cell damage and a slower wound healing process in scratched monolayer observed in our study is in accord with previously published data suggesting that DHA and short-chain trioses react with proteins by Maillard-type reaction thus inducing DNA damage, cell-cycle block, and apoptosis (35, 36, 38).

Usually, what it is detected *in vitro* may not be necessarily translate in the *in vivo* environment, but in this case it is the clinical improvement observed in the patients treated with the US-made VSL#3 and the concomitant reduction of the amount of fecal DHA that strength the *in vitro* observations. The dysruption of an appropriate mucosal barrier function and a perturbation of the immune response could be the related to some extent to an abnormal DHA production and metabolization. Considering the chronic intestinal epithelial damage and alterations in the profile of the fecal flora of HIV+ subjects, the above *in vitro* observations are therefore clinically relevant and raise concerns about the Italy-made VSL#3, confirming what anticipated in animal models of IBD by Biagioli et al. (32).

In conclusion, we have set up a new method to confirm the biosimilarity and interchangeability between different probiotic formulations especially if aimed at the dietary management of serious medical conditions. This is an area of research still unexplored but with potential serious implications for the health of the patients. In the specific case of the VSL#3 product, according to our data and previously published findings (18, 31, 32), the change of manufacturing compromised some of the biochemical and immunological properties of the product. On this matter further investigations are in progress to fully evaluate how the change in manufacturing influenced the geno and phenotype of the bacterial cells in response to their environment.

## Ethics Statement

The study was approved by the institutional review board (Department of Public Health and Infectious Diseases, Sapienza University of Rome; and the Ethics Committee of Umberto I General Hospital, Rome), number protocol 2970. All study participants signed written informed consent.

## Author Contributions

PM and DC: carried out clinical microbiology analysis, elaborate statistic of microbiology data; LL: carried out metabolomic studies, elaborate statistic of metabolomic data; BV and CP: carried out *in vitro* fermentations of probiotic formulations; VT: clinical supervision of the patients; IG: carried out cell tissue and ME studies; CD: conceived the study and wrote the manuscript.

## Conflict of Interest Statement

CD owns one share of VSL Pharmaceuticals Inc., and served in the past as Director and/or Officer of VSL Inc., Actial Farmaceutics Ltd, CD Investments Srl, CD Pharma India. He is the inventor of high concentration multistrain probiotic formulations. The VSL#3 brand is property of Actial Srl, Italy. All other authors declare that the research was conducted in the absence of any commercial or financial relationships that could be construed as a potential conflict of interest.
